# Supplementary material for: Scrutinizing the Impact of Alternating Electromagnetic Fields on Molecular Features of the Model Plant Arabidopsis thaliana
Source: Int J Environ Res Public Health. 2022 Apr 23;19(9):5144. doi: 10.3390/ijerph19095144 (PMC9099453; doi:10.3390/ijerph19095144)
Supplement: Supplementary file 1 [file ijerph-19-05144-s001.zip › Figures S1-S6.pdf]

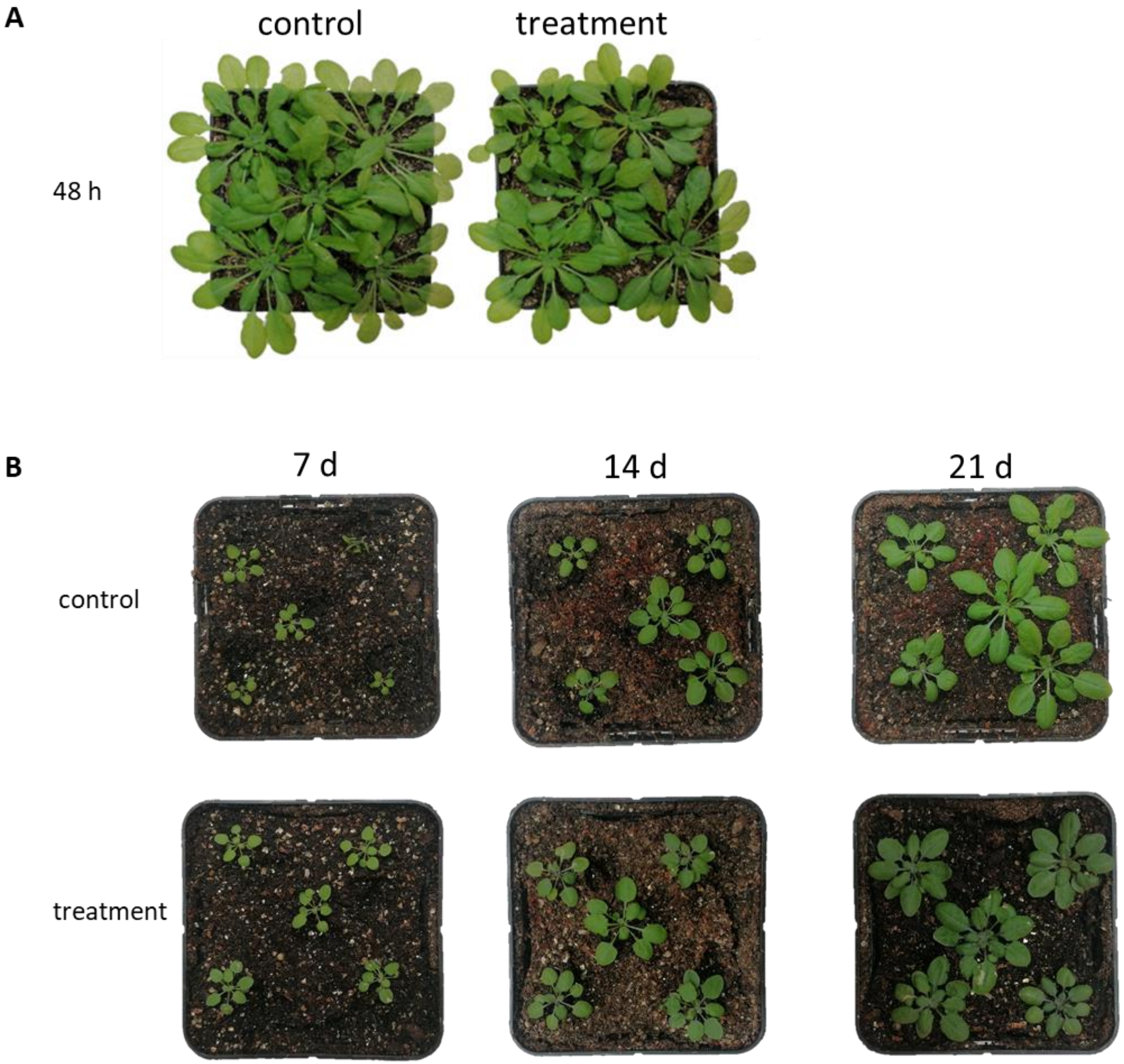

Figure S1. Phenotypical analysis of treated (treatment) and untreated (control) *Arabidopsis thaliana* (Col-0) plants, which grown on soil under optimal climate conditions. A) 30 d old A.t. plants, after 48h of treatment. **B)** Long-term treatment over 21d. Representative results of n = 3.

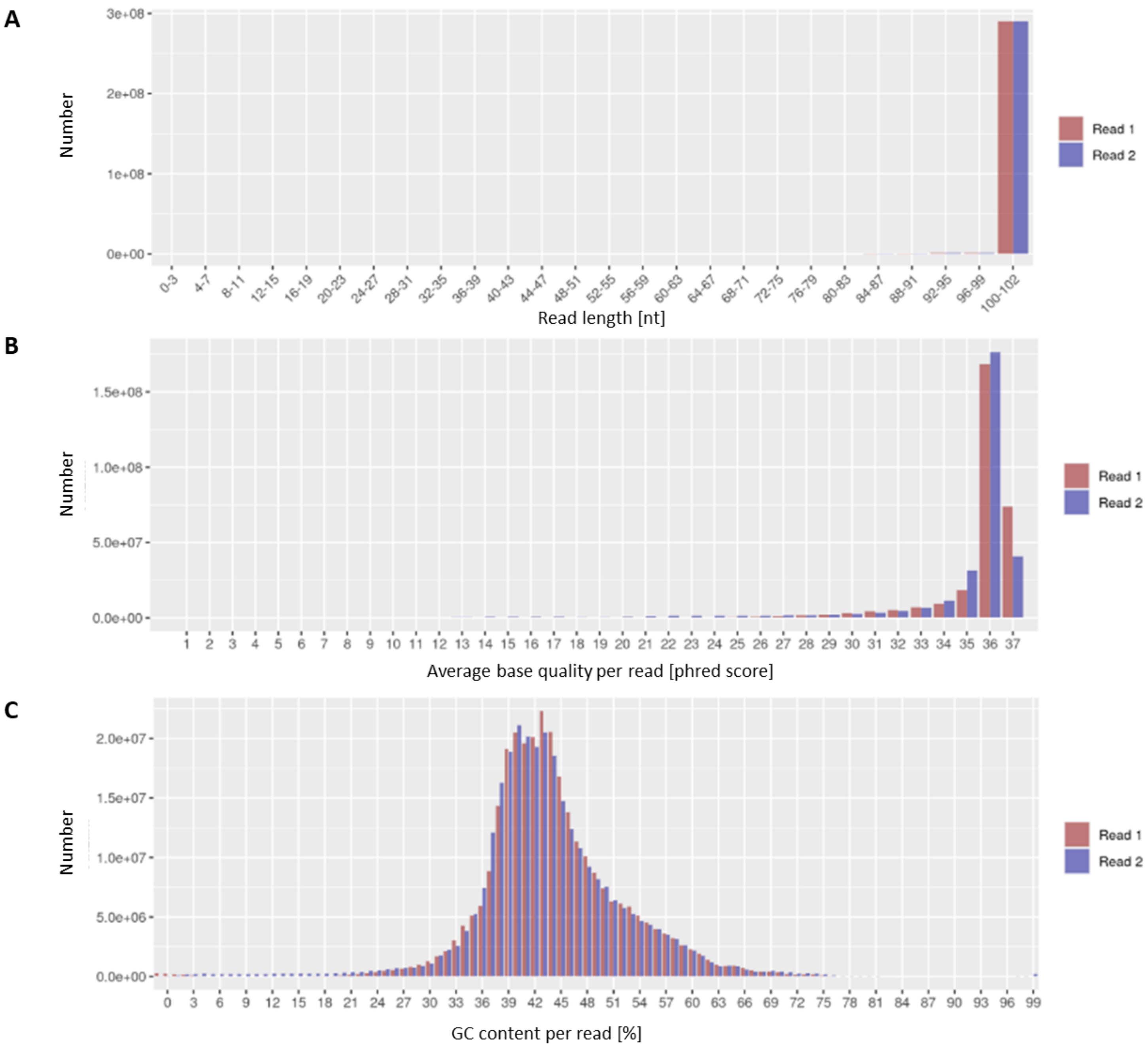

Figure S2. Raw reads. A) Sequence lengths of the trimmed FASTQ reads (average over all samples, RNA). **B)** Sequence qualities of the trimmed FASTQ reads (average over all samples; RNA). **C)** GC content of the trimmed FASTQ reads (average over all samples; RNA).

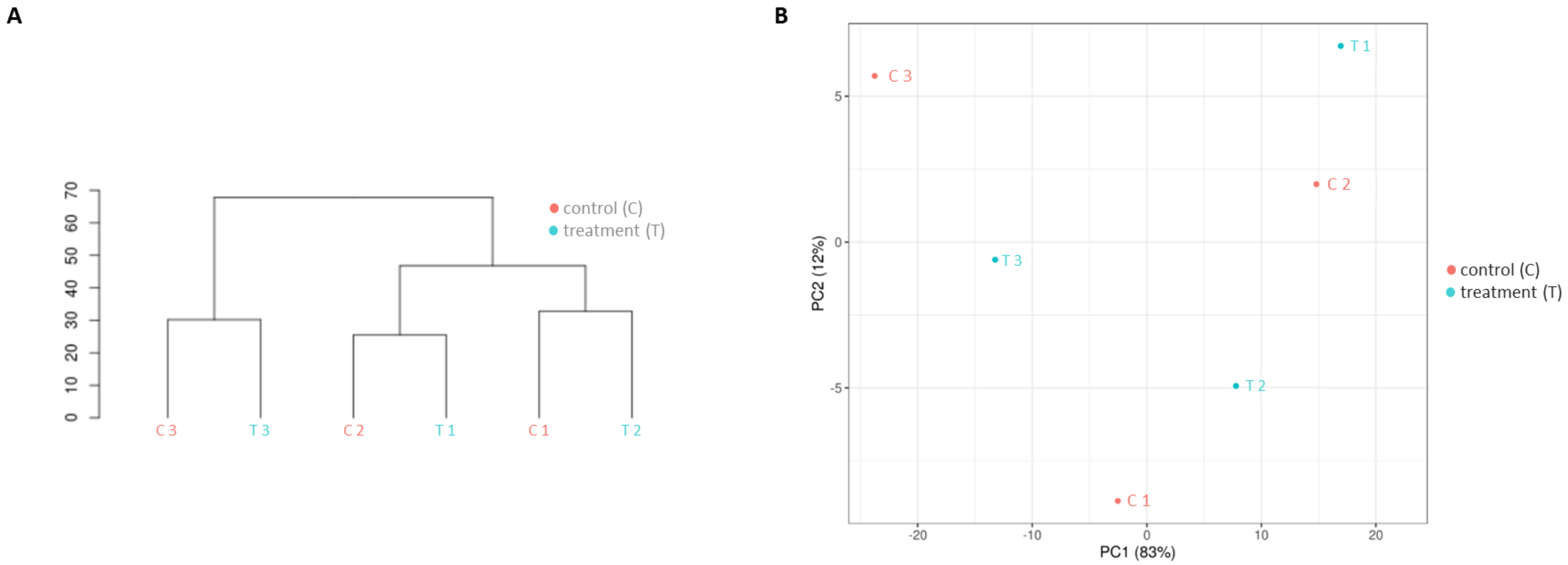

Figure S3. Analysis of expression data. Samples are colored according to group. Group T are the treatment samples and group C are the control samples, for each group are three replicates. A) Hierarchical clustering of the samples according to the similarity of their expression data based on al genes that have received at least one read in at least one sample. The Euclidean distance of the rlog-transformed data was used to calculate the distances between the samples. Samples are stained by group. B) Principal component analysis of the rlog-transformed expression data of all genes with received at least one read in at least one sample. The percentages on the axes describe the proportion of this principal component in the total variance.

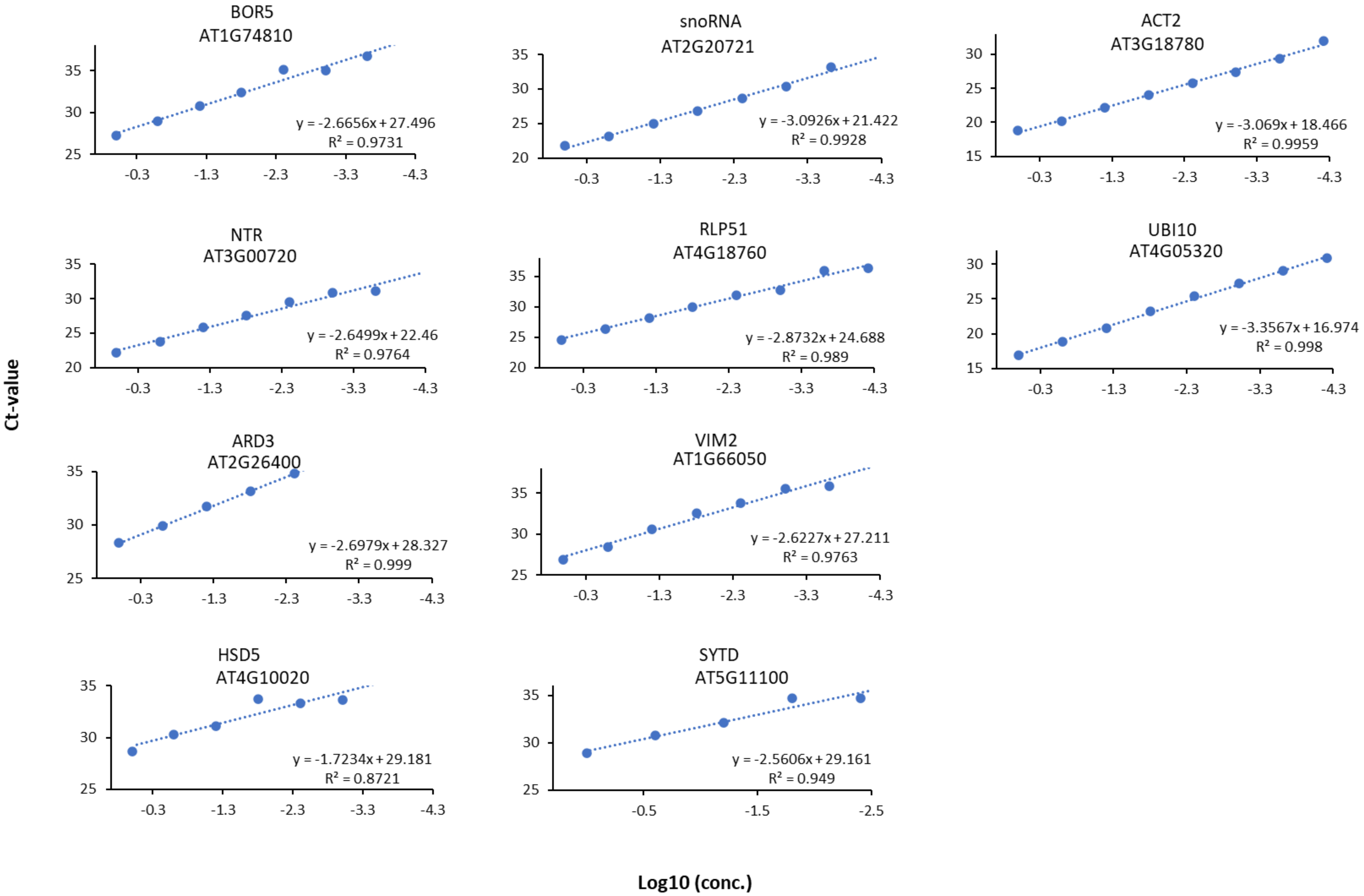

Figure S4. Series of cDNA concentration for checking the qPCR-primer system. Each primer system was tested at different cDNA concentrations in order to check the efficiency, the specificity and the linear range of the primers. The elongation time and annealing temperature were 45 seconds and 60 °C., respectively, for all systems. The ct-value is plotted against the decadic logarithm of the dilution level, n =3.

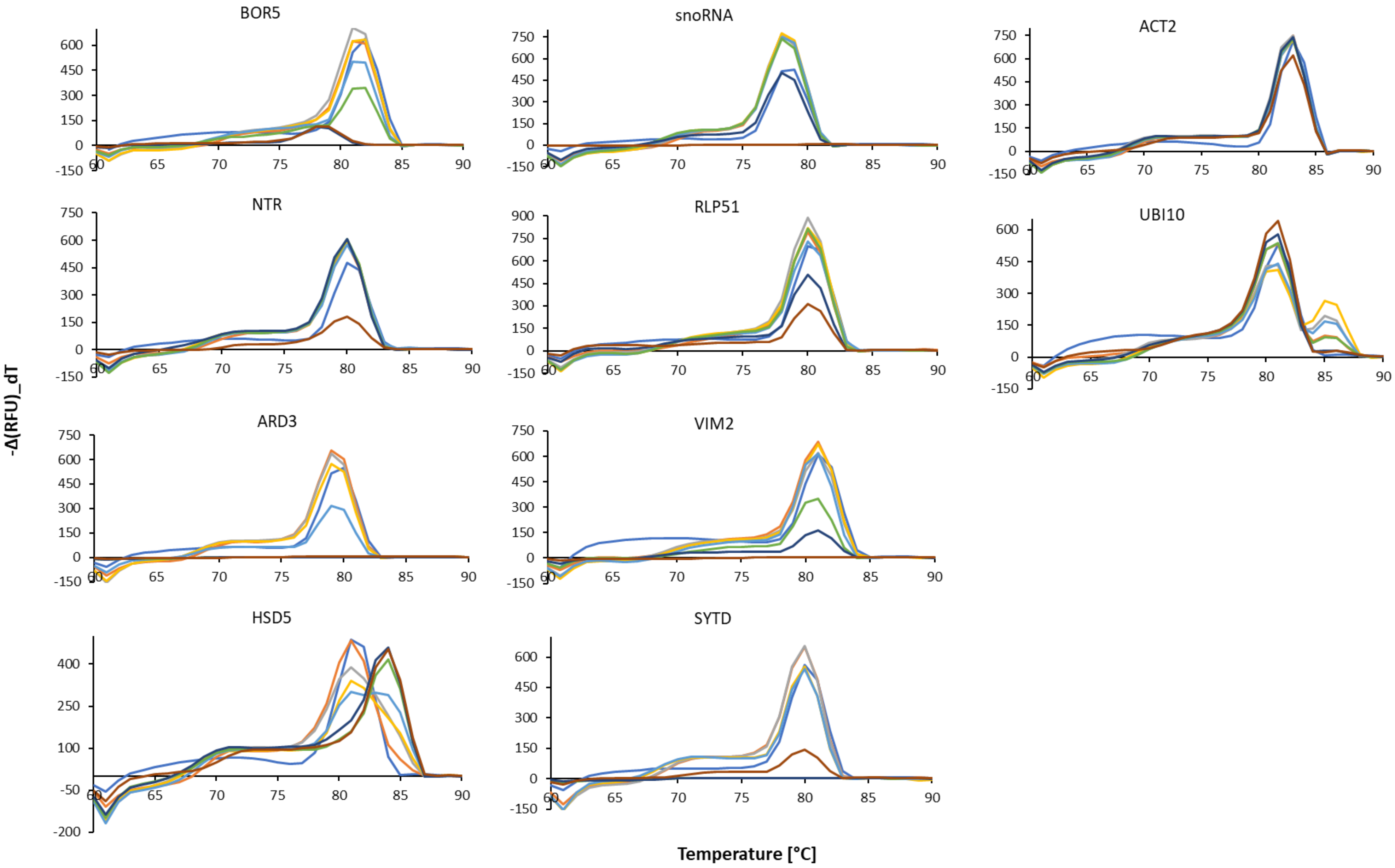

Figure S5. Melting curves of the primers for checking the PCR products. A melting curve was recorded at the end of each qPCR. A range from 60 °C to 90 °C was covered. The negative, first derivative of the relative fluorescence (-Δ(RFU)_dT) is plotted against the temperature, n = 3.

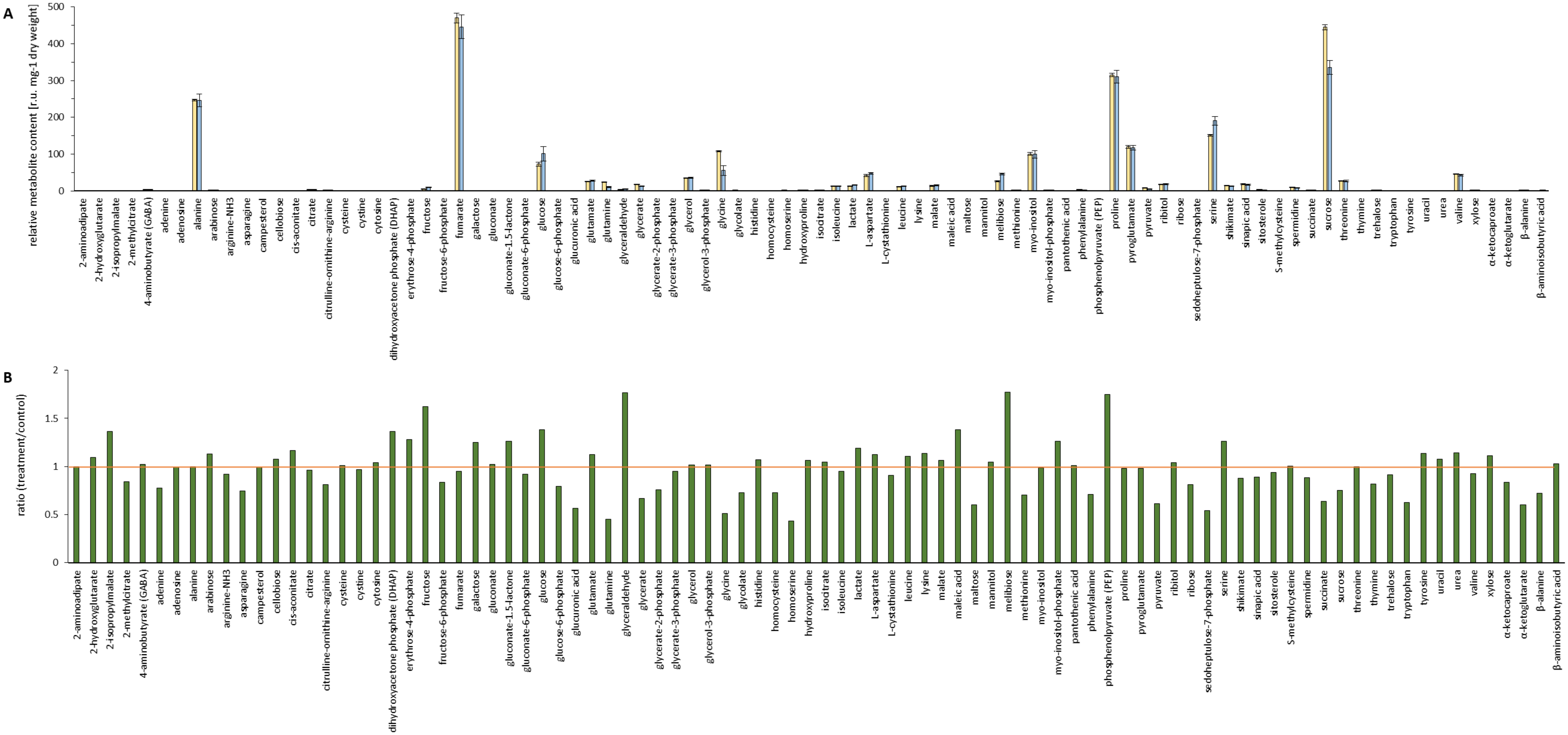

Figure S6. Metabolite profiling in *Arabidopsis thaliana* plants. In 30-day-old plants (Col-0), after 48 h of EMF irradiation, the relative metabolite levels of treatment and control extracts were measured using GC-MS. A) Overview of detected metabolites with unchanged leaf contents. B) Ratio of the relative metabolite content between treated and control extract, for a more detailed assessment of the metabolites. Independent replicates n = 3 for each treatment and control, means ± SE.
