# Supplementary figures and images for: Scrutinizing the Impact of Alternating Electromagnetic Fields on Molecular Features of the Model Plant Arabidopsis thaliana
Source: Int J Environ Res Public Health. 2022 Apr 23;19(9):5144. doi: 10.3390/ijerph19095144 (PMC9099453; doi:10.3390/ijerph19095144)

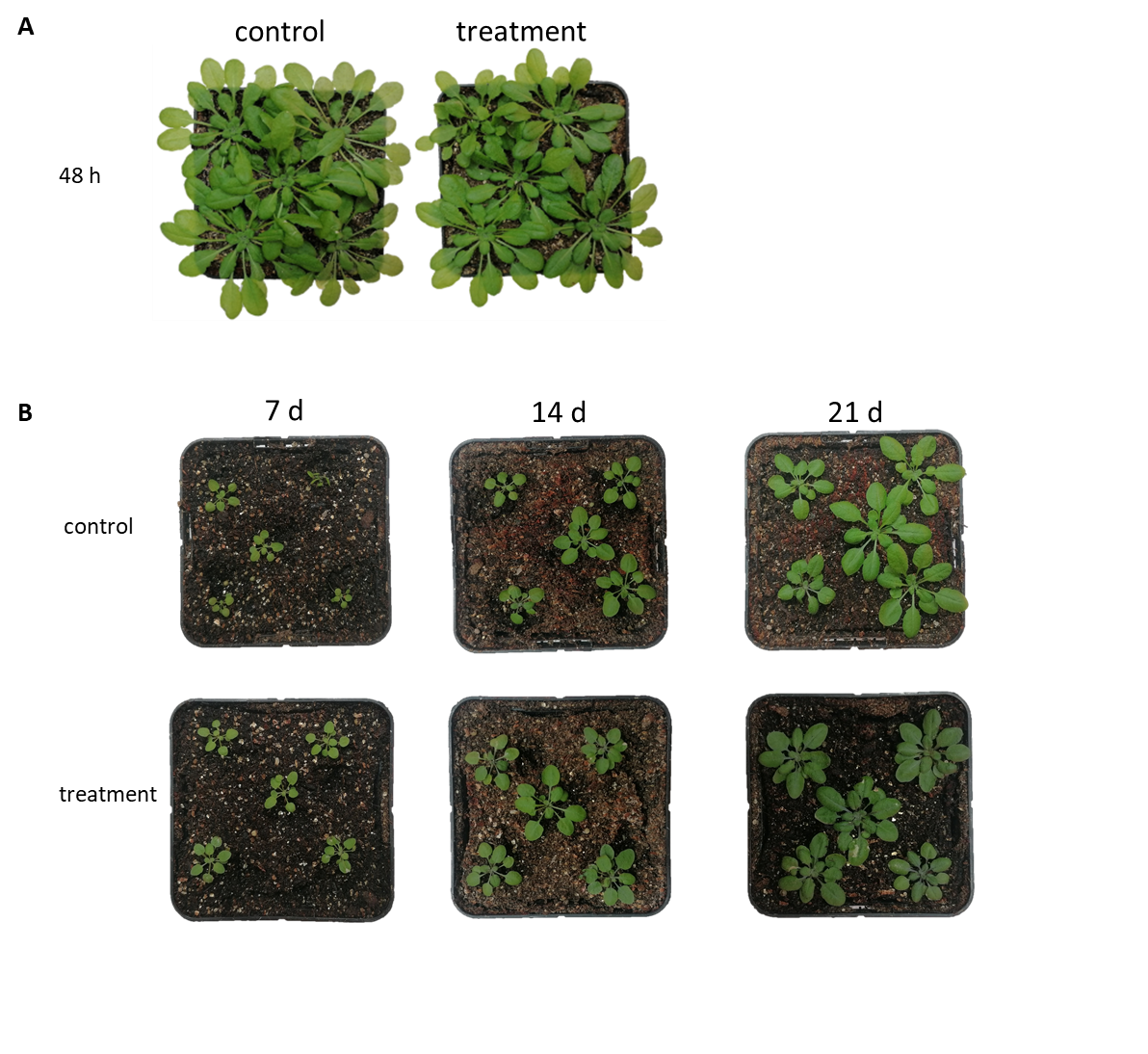

Supplement: Supplementary file 1 [file ijerph-19-05144-s001.zip › Figures S1 - S6-tiff/Figure S1.tiff]

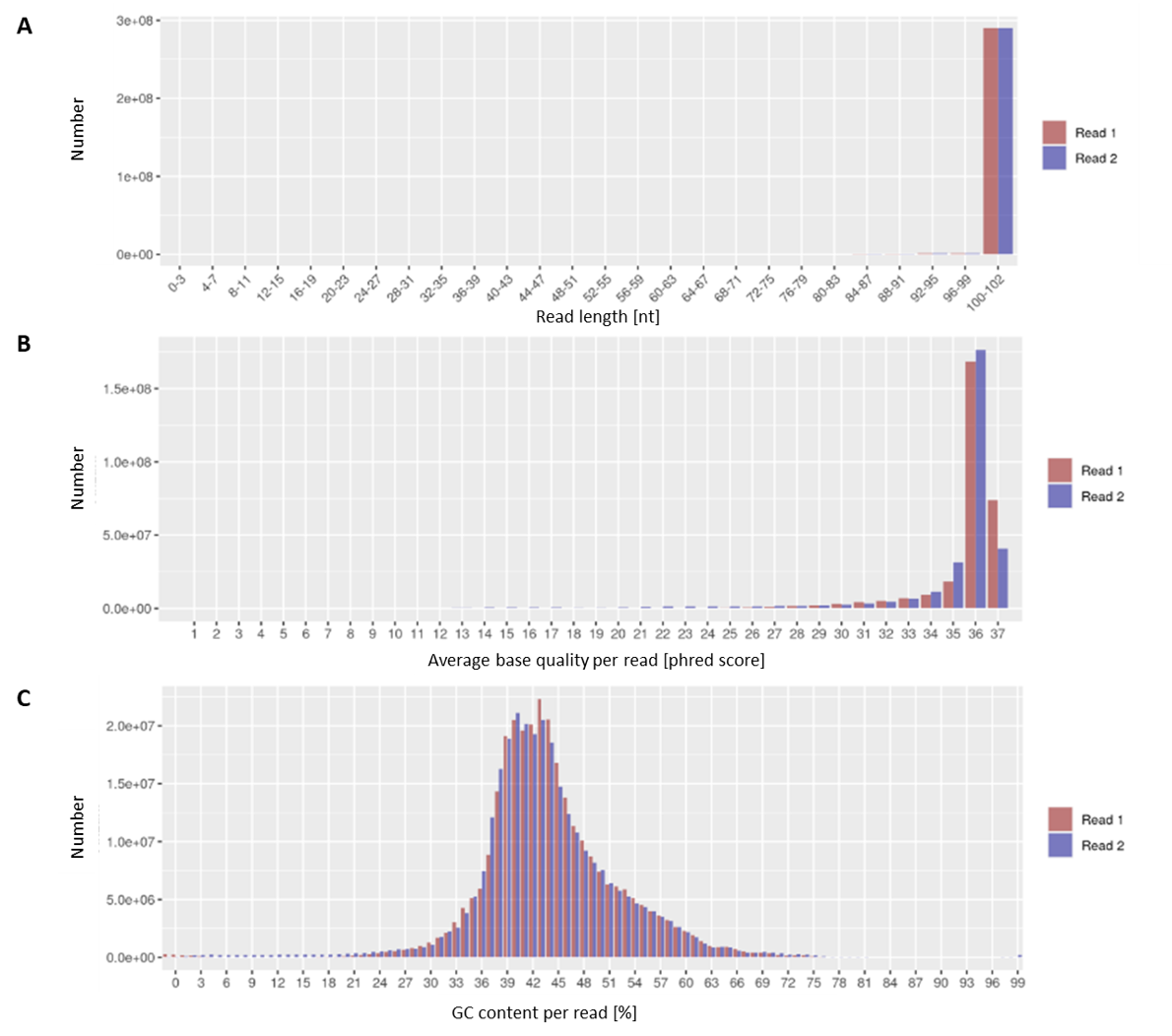

Supplement: Supplementary file 1 [file ijerph-19-05144-s001.zip › Figures S1 - S6-tiff/Figure S2.tiff]

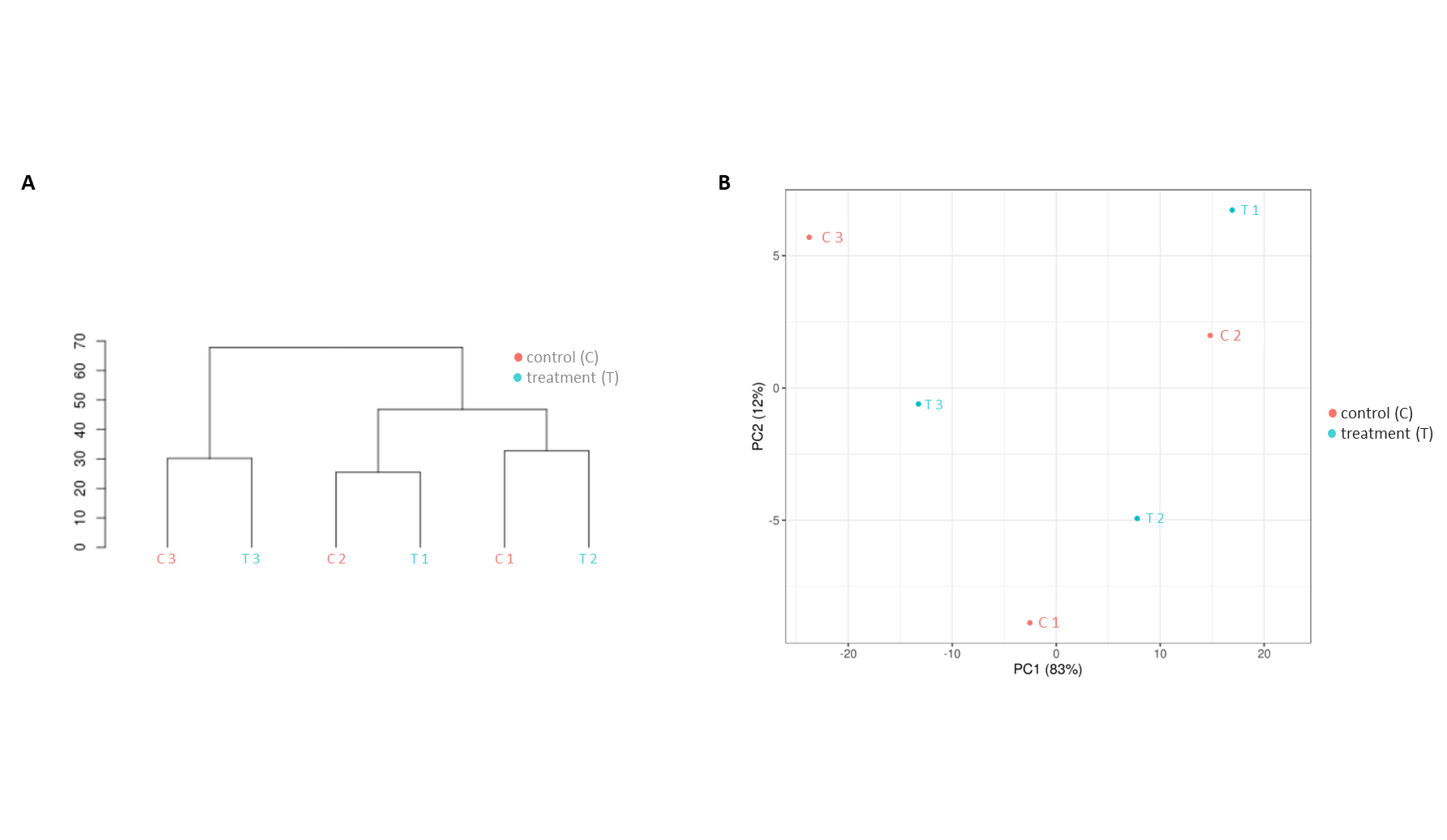

Supplement: Supplementary file 1 [file ijerph-19-05144-s001.zip › Figures S1 - S6-tiff/Figure S3.tiff]

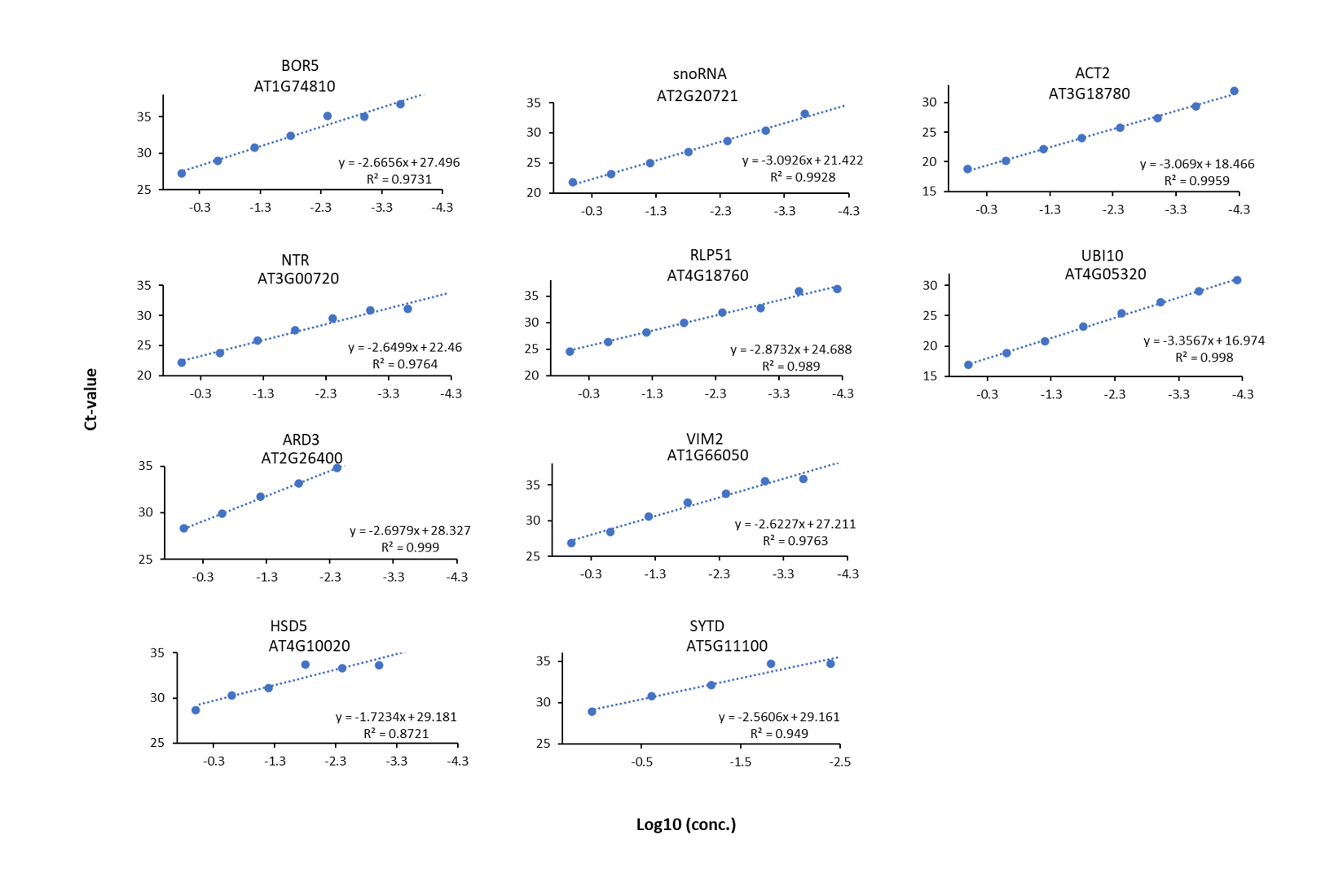

Supplement: Supplementary file 1 [file ijerph-19-05144-s001.zip › Figures S1 - S6-tiff/Figure S4.tiff]

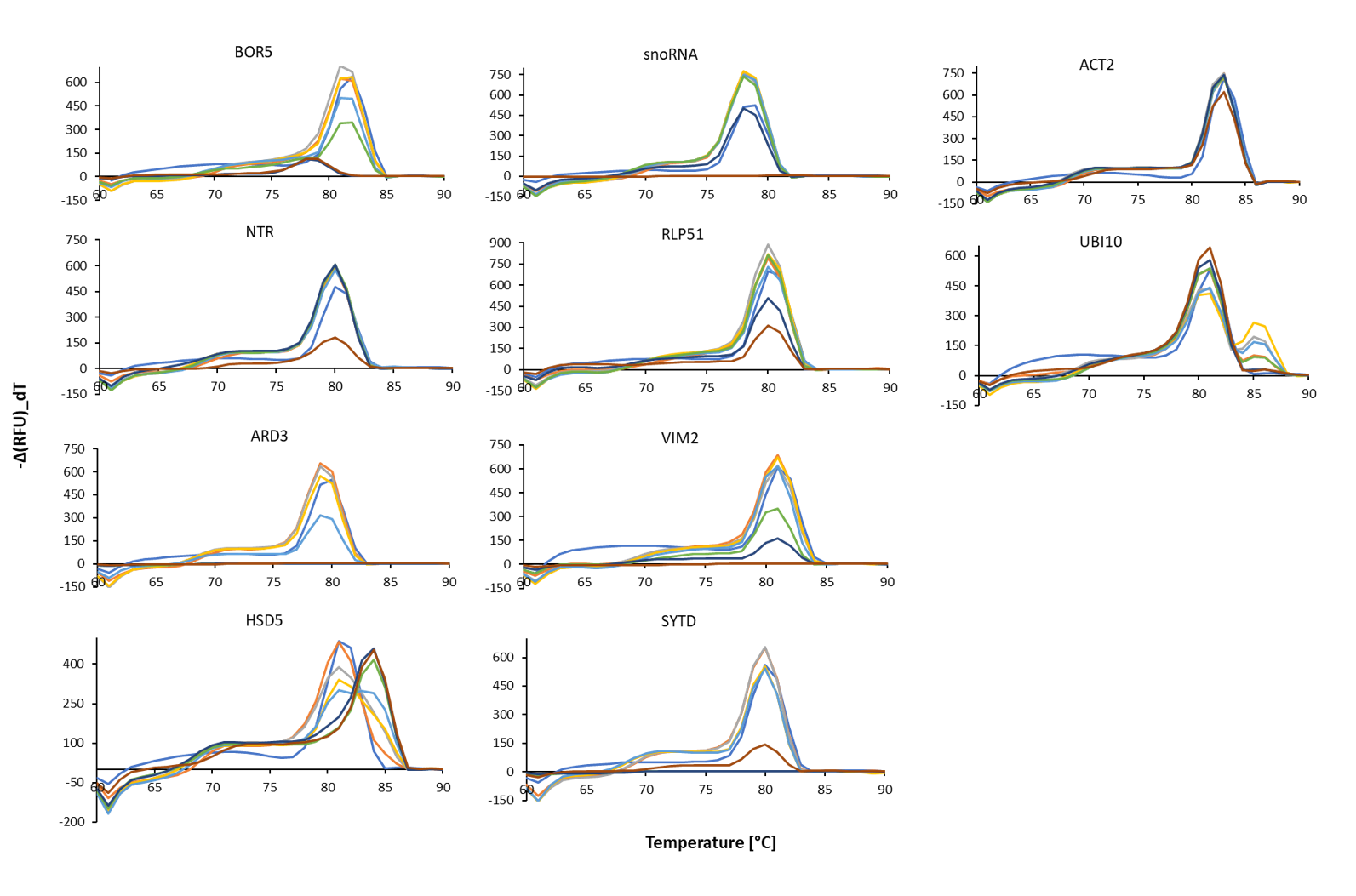

Supplement: Supplementary file 1 [file ijerph-19-05144-s001.zip › Figures S1 - S6-tiff/Figure S5.tiff]

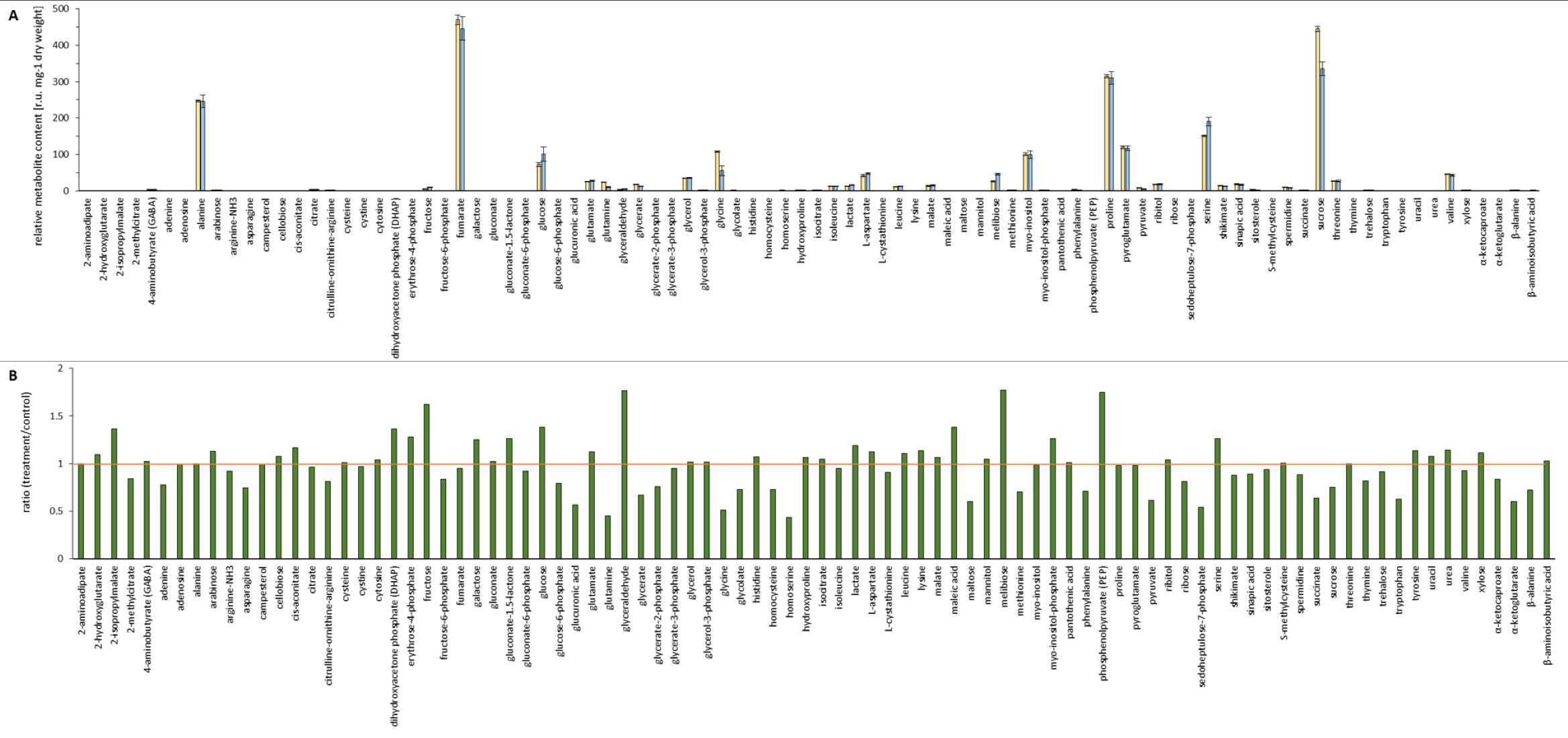

Supplement: Supplementary file 1 [file ijerph-19-05144-s001.zip › Figures S1 - S6-tiff/Figure S6.tiff]
